# Treatment Outcomes after Postoperative Radiotherapy in Triple-Negative Breast Cancer: Multi-Institutional Retrospective Study (KROG 17-05)

**DOI:** 10.3390/jpm14090941

**Published:** 2024-09-04

**Authors:** Jin Hee Kim, Sang Jun Byun, Myeongsoo Kim, Kyung Hwan Shin, Dong Yun Kim, Han Byoel Lee, Tae Hyun Kim, Yeon Joo Kim, Yong Bae Kim, Jee Suk Chang, Kyubo Kim, Sun Young Lee

**Affiliations:** 1 Department of Radiation Oncology, Dongsan Hospital, Keimyung University School of Medicine, 1035 Dalgubeol-daero Dalseo-gu, Daegu 42601, Republic of Korea; kryph@dsmc.or.kr (S.J.B.); mskim@dsmc.or.kr (M.K.); 2 Department of Radiation Oncology, Seoul National University College of Medicine, Seoul 03080, Republic of Korea; radiat@snu.ac.kr (K.H.S.); yunny0106@gmail.com (D.Y.K.); 3 Department of Radiation Oncology, Chung-Ang University Hospital, Seoul 06973, Republic of Korea; 4 Department of Surgery, Seoul National University College of Medicine, Seoul 03080, Republic of Korea; hblee80@gmail.com; 5 Department of Radiation Oncology, Proton Therapy Center, National Cancer Center, Goyang 10408, Republic of Korea; k2onco@ncc.re.kr (T.H.K.); yjkim1785@ncc.re.kr (Y.J.K.); 6 Department of Radiation Oncology, Yonsei University College of Medicine, Seoul 03722, Republic of Korea; ybkim3@yumc.yonsei.ac.kr (Y.B.K.); changjeesuk@yuhs.ac (J.S.C.); 7 Department of Radiation Oncology, Ewha Womans University School of Medicine, Seoul 07804, Republic of Korea; kyubokim@snu.ac.kr; 8 Department of Radiation Oncology, Seoul National University Bundang Hospital, Seoul 03080, Republic of Korea; 9 Department of Radiation Oncology, Chonbuk National University Hospital, Jeonju 54907, Republic of Korea; sylee78@jbnu.ac.kr

**Keywords:** triple-negative breast neoplasms, radiotherapy, failure pattern, survival, prognostic factors

## Abstract

Background: We designed a multi-institutional retrospective study to investigate the previously unreported failure pattern, survivals, and prognostic factors after postoperative radiotherapy (PORT) in triple negative breast cancer (TNBC) patients in South Korea. Materials and Methods: We retrospectively reviewed 699 patients with TNBC who underwent PORT at six institutions between 2008 and 2010. The median follow-up period was 94 months (range: 7–192 months). There were 216, 380, and 100 patients in stages I, II, and III, respectively. Results: After 94 months post-treatment, all patients with pathologic complete remission after neoadjuvant chemotherapy were alive without any failure. Distant metastasis was the main cause of failure. The 5-year overall survival rate was 91.4%, 5-year loco-regional relapse-free survival rate (LRRFS) was 92.3%, 5-year distant metastasis-free survival rate (DMFS) was 89.4%, and 5-year disease-free survival rate (DFS) was 85.2%. On multivariate (Cox) analysis, T and N stages were significant prognostic factors for survival, and lympho-vascular invasion (LVI) was a significant factor for LRRFS and DMFS. *Ki-67* expression was significantly associated with LRRFS and DFS. Conclusion: We verified that T and N stages, LVI, and *Ki-67* expression were significantly associated with survival outcomes after PORT in TNBC.

## 1. Introduction

Triple-negative breast cancer (TNBC) accounts for 10–15% of all breast cancers and the clinical course of TNBC and the risk factors are known to be different from those of estrogen receptor (ER)-positive cancers [1]. TNBCs lack estrogen and progesterone receptors and express low levels of human epidermal growth factor receptor 2 (HER2). Therefore, they do not respond to hormonal therapy or anti-HER2 therapies. TNBC is the most malignant subtype of breast cancer with a poor prognosis [1]. Clinically, they are characterized by younger age at initial diagnosis, greater frequency in African-American women, large tumor size at diagnosis, a high histological grade, and high frequency of lymph node involvement [2]. They also show an aggressive clinical course with a predominance of involvement of the visceral organs, mainly the lungs, central nervous system, and lymph nodes [2,3].

While TNBC is widely believed to be particularly lethal, most patients with early-stage TNBC never experience distant metastasis or die of breast cancer. However, the survival time of patients with metastatic TNBC is notably shorter than that of patients with metastatic ER-positive breast cancers [3]. Immunotherapy has recently emerged as an effective treatment option for TNBC. Chemoimmunotherapy is emerging as the standard of care for early and advanced stages of TNBC [4,5]. As novel therapeutic targets demonstrate efficacy and immunomodulatory effects in TNBC, combination strategies are being explored, immune check-point inhibitor (ICI) monotherapy is emerging, and radiotherapy with immunomodulatory effects is expected to play a role in TNBC [6]. To date, there have been no reports on failure patterns, survivals, and prognostic factors in Korean patients with TNBC who underwent postoperative radiotherapy (PORT).

This was a multi-institutional retrospective study to investigate failure patterns, survivals, and prognostic factors after PORT of patients with TNBC in South Korea which has not been previously reported.

## 2. Materials and Methods

### 2.1. Study Population

Medical records of patients with TNBC who met the inclusion and exclusion criteria at six institutions in South Korea between January 2008 and December 2010 were reviewed and analyzed retrospectively. The median follow-up period was 94 months (range: 7–192 months). We used the following as inclusion criteria: (1) patients with operable breast cancer who were treated with breast-conserving surgery (BCS) or mastectomy with curative intent, and (2) patients with TNBC who underwent PORT. Patients with distant metastases or previous history of other cancers were excluded. Clinical records, including patient age; pathologic diagnosis; stage; type and date of surgery; site, dose, and duration of radiotherapy; chemotherapy regimen and duration of administration; response to neoadjuvant chemotherapy; survival; date and treatment of recurrence and metastasis; and follow-up period, as well as pathological information such as histologic subtype, histologic grade (HG), lympho-vascular invasion (LVI), extracapsular extension status (ECE), and *Ki-67* were retrieved from the reports of each institution.

### 2.2. Statistical Analysis

Local recurrence was defined as tumor recurrence in the ipsilateral breast or chest wall, and regional recurrence was defined as that in the ipsilateral axillary, supraclavicular, and/or internal mammary nodes. Distant metastases were defined as disease metastases other than local and/or regional recurrence (LRR). Loco-regional recurrence-free survival (LRRFS) was defined as the time from the initiation of treatment to the first LRR. The time interval between the start of treatment and distant metastasis was measured as distant metastasis-free survival (DMFS). Disease-free survival (DFS) was defined as the time from the start of treatment to the date of relapse, death, or the last follow-up. Overall survival (OS) was defined as the time from treatment initiation to death from any cause. The time interval between the start of treatment and death due to breast cancer was measured as cause-specific survival (CSS). Categorical variables were compared using the chi-square test, and continuous variables were compared using the *t*-test or Mann–Whitney U test. Actuarial survival rates were calculated using the Kaplan–Meier method. The log-rank test (*p* is less than 0.05 has statistical significance) was used for univariate analysis, and the Cox proportional-hazard model was used for multivariate analysis, incorporating factors with a *p*-value < 0.1 on univariate analysis. All statistical analyses were performed using the PAWS Statistics for Windows ver. 18.0 (SPSS Inc., Chicago, IL, USA).

## 3. Results

### 3.1. Characteristics

A total of 669 patients from six institutions were included (Table 1).

The median patient age was 49 years (range: 24–80 years). Pathologically, 622 patients (88.9%) had invasive ductal carcinoma. Histological, 538 (77%), 103 (14.7%), and 10 (1.4%) patients were grades 3, 2, and 1, respectively. LVI was reported in 240 patients (34.3%) and ECE was reported in 49 patients (7%). *Ki-67* was reported negative in 3, <5% in 131 patients, 6–25% in 211 patients, and >26% in 198 patients. According to the tumor size, 268 patients (38.3%) were T1, 355 patients (50.8%) were T2, and 61 patients (8.7%) were T3. There were 254 patients (36.3%) who clinically had axillary lymph node metastases. According to American Joint Committee on Cancer (AJCC) staging 7th edition, there were 3 patients (0.4%) in stage 0, 216 (30.9%) in stage I, 270 (38.6%) in stage IIA, 110 (15.7%) in IIB, and 100 (14.3%) in stage III (Table 1)

### 3.2. Treatments

Neoadjuvant chemotherapy was administered to 130 patients (18.6%), mainly with an adriamycin-cyclophosphamide (AC) regimen (70.8%). Breast-conserving surgery and mastectomy were performed in 634 (90.7%) and 65 patients (9.3%), respectively (Table 2).

Most patients were irradiated 45–50.4 Gy with 25–28 fractions to the breast or chest wall followed by a tumor bed boost with 10–16 Gy with 5–8 fractions. Simultaneously, additional radiation fields were irradiated to the supraclavicular or internal mammary lymph node areas related to the stage and postoperative pathological results, mainly with 45–50 Gy in 25 fractions. Eighty patients were treated with hypofractionation radiotherapy (RT), mainly 39 Gy in 13 fractions, with a boost of 9 Gy in 3 fractions at the tumor bed. Adjuvant chemotherapy was performed in 596 patients (85.3%); the most common regimen was 5-fuorouracil-adriamycin-cyclophosphamide in 259 patients (43.4%), followed by AC in 118 patients (19.8%), and AC-paclitaxel regimen in 68 patients (11.4%). AC-docetaxel was administered to 46 patients, cyclophosphamide-methotrexate-5-fuorouracil to 44 patients, and docetaxel-adriamycin to 35 patients (Table 2).

### 3.3. Outcomes and Prognostic Factors

With a median follow-up of 94 months (range: 7–192) after treatments, including surgery, radiation therapy, and/or chemotherapy (neoadjuvant or adjuvant), 594 patients (85.0%) had no evidence of disease, 31 patients (5.0%) were alive with disease, 3 patients (0.4%) died due to other causes, and 67 (9.6%) died from the breast cancer.

After neoadjuvant chemotherapy was performed in 130 patients, pathological complete remission (pCR) was reported in 11 patients (8.4%); 56 patients (43%) showed pCR of primary tumor. All patients who achieved a pCR after neoadjuvant chemotherapy survived without recurrence or distant metastasis.

**Figure 1:**
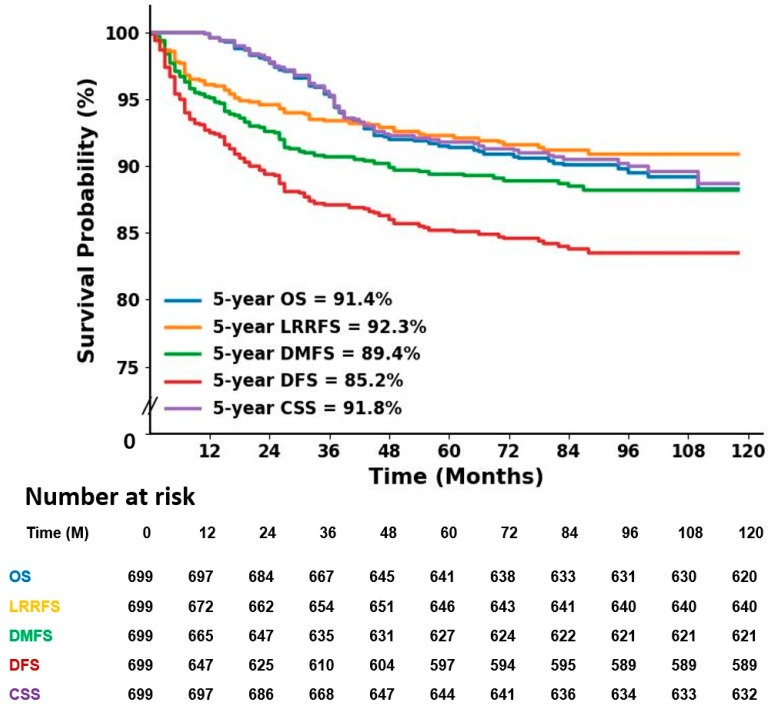
Kaplan–Meier survival curves of all patients. CSS, cause-specific survival; DFS, disease-free survival; DMFS, distant metastasis-free survival; LRRFS, loco-regional relapse-free survival; OS, overall survival.

The 5-year OS was 91.4%, 5-year LRRFS was 92.3%, 5-year DMFS was 89.4%, 5-year DFS was 85.2%, and 5-year CSS was 91.8% (Figure 1).

**Figure 2:**
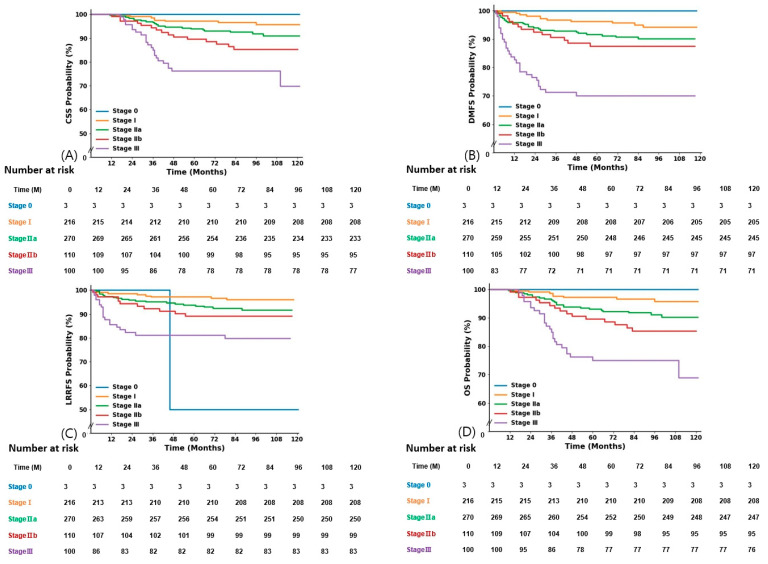
Kaplan–Meier survival curves by stage. CSS, cause-specific survival (**A**); DMFS, distant metastasis-free survival (**B**); LRRFS, loco-regional relapse-free survival (**C**); OS, overall survival (**D**).

According to the stage, the 5-year OS, 5-year CSS, 5-year LRRFS, and 5-year DMFS were 100%, 100%, 50%, and 100% for stage 0, 97.2%, 97.2%, 97.2%, and 96.2% for stage I, 93.1%, 93.9%, 93.8%, and 91.6% for stage IIA, 89.6% 89.6%, 89.2%, and 87.5% for stage IIB, and 75.1%, 76.3%, 81.2%, and 70.1% for stage III, respectively (Figure 2).

In univariate analysis, age <50 years, T stage, N stage, HG, LVI, ECE, and *Ki-67* were statistically significant prognostic factors for survival, including OS, LRRFS, DMFS, DFS, and CSS (Table 3).

In the multivariate analysis, T and N stages were statistically significant factors related to all survival including OS, LRRFS, DMFS, DFS, and CSS (Table 4).

LVI (hazard ratio (HR), 1.227; 95% confidence interval (CI): 1.00–1.49, *p* = 0.04) and *Ki-67* (>5%, HR, 3.148; 95% CI: 1.246–7.955, *p* = 0.015) were a statistically significant factor for LRRFS. LVI (HR, 0.6; 95% CI: 0.36–1.0, *p* = 0.05) was a statistically significant factor for DMFS. *Ki-67* (>5%, HR, 2.188; 95% CI: 1.14–4.197, *p* = 0.019) was a statistically significant factor for DFS (Table 4).

### 3.4. Patterns of Failure

**Figure 3:**
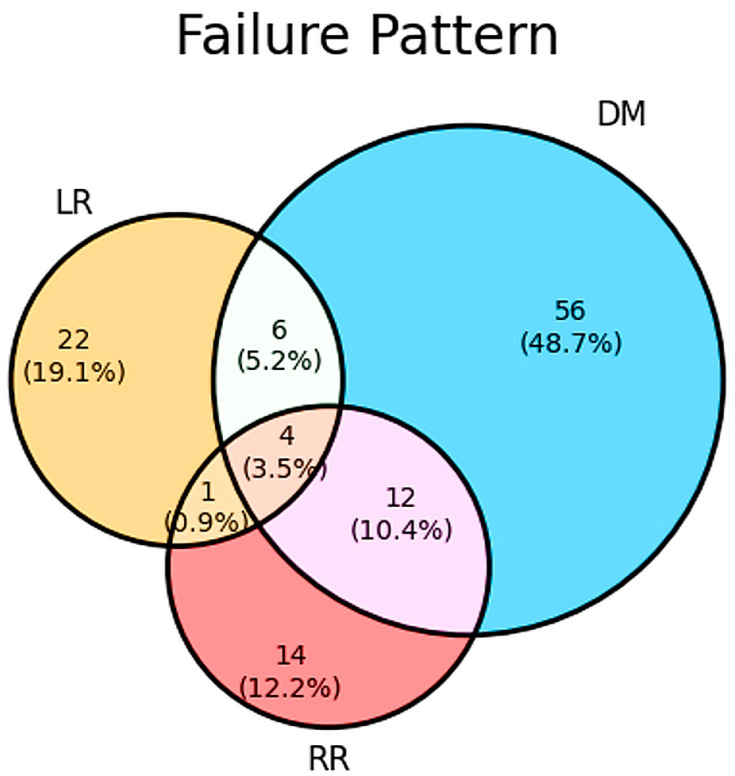
Patterns of failure. DM, distant metastasis; LR, local recurrence; RR, regional recurrence.

Treatment failure was observed in 115 patients (16.45%) and distant metastasis (78 patients, 11.2%) was the main failure pattern. Local recurrence was reported in 33 patients (4.7%) and regional recurrence was in 31 patients (4.4%). There were 6 cases of distant metastasis and local recurrence, 12 cases of distant metastasis and regional recurrence, 4 cases of distant metastasis and local and regional recurrences, and 1 case of local and regional recurrences. The main failure pattern was distant metastasis 67.8% (78/115) of all patients with failure and included all cases of simultaneous recurrence; local and regional recurrences were 28.7% (33/115) and 26.9% (31/115) of all failures, respectively (Figure 3).

The median times of distant metastasis and local and regional recurrences were 15 months (range: 1–87 months), 11 months (range: 1–79 months), and 15 months (range: 1–88 months), respectively. The most frequent sites of distant metastasis were the lung (*n* = 38), bone (*n* = 25), brain (*n* = 20), liver (*n* = 14), mediastinal lymph node (*n* = 12), contralateral neck and axillary lymph node (*n* = 8), followed by abdominal lymph node (*n* = 3), adrenal gland (*n* = 1), skin (*n* = 1), ovary (*n* = 1), and the pleura (*n* = 1).

## 4. Discussion

TNBC is a heterogeneous subgroup of invasive breast carcinomas characterized by the lack of ER, progesterone receptor, and HER2 [1]. Recently, TNBC has been characterized as having ≤1% cellular expression of ER and progesterone receptor as determined by immunohistochemistry (IHC) and having *HER2* expression of 0 to 1+ by IHC, or 2+ by IHC and fluorescence in situ hybridization negative (i.e., not an amplified gene copy number), according to the American Society of Clinical Oncology/College of American Pathologists guidelines [7,8].

TNBC is more prevalent in younger women (<50 years) and in carriers of deleterious germline mutations in defined susceptibility genes, including breast cancer types 1 and 2 [3]. In addition, the median age of patients in this was 49 years. Compared to other breast cancer subtypes, TNBC usually displays an aggressive disease course and dismal prognosis, regardless of race, age, and stage of presentation. TNBC is characterized by an early tendency to metastasize, a higher recurrence rate, and worse survival; nevertheless, it is significantly more sensitive to chemotherapy than other breast cancer subtypes [9]. For most patients with early-stage TNBC, sequential anthracycline- and taxane-based NACT represents the standard therapeutic approach, with pCR strongly correlating with long-term survival outcomes [10]. In this study, the neoadjuvant chemotherapy regimen was mostly a sequential anthracycline and taxane regimen and all patients who showed pCR after neoadjuvant chemotherapy survived without recurrence or metastasis. A neoadjuvant study involving the administration of chemotherapy before surgery suggested that this treatment is very effective in a minority of women with TNBC who have a complete pathologic response and thus an excellent outcome; in contrast, the outcome for most patients with residual disease after treatment is relatively poor [3].

The principles of local therapy, such as surgery and radiation, are applied similarly for all breast cancer subtypes, and there are no TNBC-specific recommendations for local management [11]. A study using the Surveillance, Epidemiology, and End Results program database between 1973 and 2014 reported that breast-conserving surgery (BCS) and RT demonstrated a better prognosis than modified radical mastectomy (MRM) alone or MRM and RT treatments for patients with early-stage TNBC [12]. In this study, BCS and RT were performed in 634 patients (90.7%), and mastectomy and RT were performed in 65 patients (9.3%). A meta-analysis of TNBC among 22 studies showed that patients who underwent BCS were less likely to develop LRR and distant metastasis than those who underwent mastectomy in the TNBC group [13]. Kim et al. [14] reported that breast-conserving therapy achieved better locoregional recurrence-free, disease-free, and overall survival rates than mastectomy in patients with pT1-2N1 TNBC. Adjuvant RT was associated with a significantly lower risk of locoregional recurrence in TNBC patients irrespective of the type of surgery [15]. Dixit et al. [16] reported that radiation treatment was significantly associated with improved outcomes in early-stage TNBC. Moran reported that TNBC is not a contraindication for breast conservation therapy [17]. In a randomized study, Wang et al. [18] reported that standard adjuvant chemotherapy plus radiation therapy was more effective than chemotherapy alone in women with triple-negative stages I and II breast cancer after mastectomy. In this study, adjuvant chemotherapy was performed in 596 patients (85.3%); the most common regimen was fluorouracil-adriamycin-cyclophosphamide in 259 patients (43.4%), followed by adriamycin-cyclophosphamide (AC) in 118 patients (19.8%), and AC-T regimen in 68 patients (7.7%). Ren et al. [19] reported that adjuvant chemotherapy improved recurrence-free survival (RFS) in patients with T1c TNBC but not in those with T1b. In addition, taxane-free and anthracycline-based regimens might be sufficient to achieve RFS benefits in T1N0M0 TNBC patients. Sharma [20] suggested that the biological heterogeneity of TNBC has hindered the development and evaluation of novel agents; however, recent advancements in sub-classifying TNBC have paved the way for further investigation of more effective systemic therapies, including cytotoxic and targeted agents.

Five-year OS was 91.4%, 5-year LRRFS was 92.3%, 5-year DMFS was 89.4%, 5-year DFS was 85.2%, and 5-year CSS was 91.8% in all patients. These results are comparable to other studies [12,13,15,21]. Van Roozendaal et al. [21] showed 5-year DFS was 78.7%, DMFS 80.5%, and 5-year OS 82.3% in a cohort study of 2546 patients with clinically T1-2N0 TNBC. Howard and Olopade [22] reported that, whereas long-term survival for stage I TNBC was similar to that of other subtypes, outcomes dramatically worsened with increasing stage, with almost half of the women dying of stage III disease within 4 years of diagnosis. Agarwal et al. [2] reported that both OS and DFS were shorter in TNBC than in non-TNBC, and in a stagewise comparison, OS differed significantly only in stage III. However, in this study, 5-year OS was 75.1%, even for stage III TNBC, unlike these studies.

T and N stages were statistically significant factors for OS, DMFS, DFS, and CSS in multivariate analysis. Leon-Ferre et al. [23] reported that in multivariate analysis, only higher nodal stage, lower TIL levels, and the absence of adjuvant chemotherapy were associated with worse invasive DFS and OS in the early stage TNBC. Dixit et al. [16] reported that the significant prognostic and predictive factors for OS were adjuvant radiation treatment, chemotherapy, T stage, lymph node involvement, and lympho-vascular space invasion (LVSI), the results were similar for breast cancer-specific survival, DMFS, LRFS, and LRRFS except that LVSI.

Li et al. [24] suggested that *Ki-67* may be an indicator of a poor prognosis in TNBC. *Ki-67* is a nuclear antigen present in proliferative cells. *Ki-67* is considered one of the most significant indicators of tumor cell proliferation. *Ki-67* expression reliably and quickly reflects the proliferation of malignant cells and is correlated with tumor size and lymph node metastasis in breast cancer but is not associated with age or clinical stage. The increased expression of *Ki-67* may predict increased proliferation of breast cancer cells, enhanced invasiveness, faster tumor growth, and a high incidence of lymph node metastases. In this study, *Ki-67* > 5% was significantly associated with poor LRRFS and DFS in multivariate analysis (Table 4). Zhu et al. [25] reported that a *Ki-67* cutoff of 30% had early independent prognostic and predictive potential for OS and DFS in TNBCs, and *Ki-67* > 30% was significantly associated with worse prognosis, especially in stage I patients. Selz et al. [26] reported that *Ki-67* > 20% was the only independent prognostic factor associated with increased LRR in patients with breast cancer having negative lymph nodes after MRM. Recently, van den Ende et al. [27] reported that high *Ki-67* expression is a biomarker associated with pCR after treatment with neoadjuvant chemotherapy in TNBC.

Ahn et al. [28] reported that LVI is a negative prognostic factor for TNBC after surgery. In this study, LVI was significantly associated with poor LRRFS and DMFS in the multivariate analysis (Table 4). Fayaz et al. [29] reported that clinical stage and LVI were the only significant prognostic factors for survival in patients with TNBC. Kennedy et al. [30] reported that pathological lymph node positivity and LVI predicted worse freedom from distant metastases in patients with residual disease after neoadjuvant chemotherapy for TNBC. In this study, the multivariate analysis showed that LVI was significantly associated with LRRFS and DMFS in patients with TNBC (Table 4).

Radosa et al. [31] reported that TNBC is more common among young females while an age stratification study showed that there was no relationship between the LRR and the age of patients with TNBC. Younger age (<50 years) was a statistically significant poor prognostic factor for DMFS and DFS in the univariate analysis. However, there was no statistically significant difference in multivariate analysis in this study. In addition, there was a report [32] that longer treatment duration was associated with poorer OS in TNBC; therefore, we analyzed it but found no relationship between longer treatment duration and survival rate.

TNBC is the most aggressive breast cancer subtype and is characterized by a substantial risk of early disease recurrence and mortality [33]. Chen et al. [34] reported that the LRR risk of TNBC was the highest, which is 3.31 times non-TNBC, in a meta-analysis of 15 studies involving 21,645 patients with invasive breast cancer after BSC. In this study, local recurrence occurred in 33 patients (4.7%), regional recurrence in 31 patients (4.4%), and distant metastasis in 78 patients (11.2%). Van Roozendaal et al. [18] also reported similar results such as 2.9% regional recurrence, 4.2% local recurrence, and 12.2% distant recurrence on 2548 patients with clinical T1-2N0 TNBC. Dent et al. [35] reported that early-stage TNBC was usually characterized by high rates of disease recurrence in the first 4 years and the pattern of recurrence was characterized by a rapidly rising rate in the first 2 years following diagnosis, a peak at 2 to 3 years followed by a decline in recurrence risk over the next 5 years, and a very low risk of recurrence thereafter, and the most women with TNBC who had no evidence of progression after 8 years did not recur thereafter. Van Roozendaal et al. [21] showed that DFS is more threatened by distant recurrence, affecting their overall survival in TNBC. Like previous studies [21,35], we observed that the main treatment failure was distant metastasis (67.8% of all treatment failures in patients). Distant metastases occurred in the following order: lung, bone, brain, and liver, with the most frequent site of distant metastasis being the lung. In this study, the median times of locoregional recurrence and distant metastasis were 11 and 15 months, and >80% of patients with treatment failure had local recurrence within 3 years and distant metastasis within 4 years.

Advances in research have markedly altered the therapeutic landscape for TNBC [36]. Major innovations in the application of immunotherapy have occurred as researchers have gained greater insight into the immunogenicity of TNBC. Chemoimmunotherapy is the standard of care for early disease regardless of Programmed *Cell Death-ligand 1 (PD-L1)* expression and PD-L1-positive advanced disease [4,5]. Initial ICI monotherapy trials have demonstrated modest but durable responses, establishing ICI as a viable treatment modality. Combination trials with chemotherapy have shown improved responses in patients with both early and advanced TNBC. Combination strategies are being explored to identify novel therapeutic targets that demonstrate efficacy and immunomodulatory effects in the treatment of TNBC. Combined treatment with radiotherapy and immunotherapy, which have immunomodulatory effects, has been reported to play a role in TNBC [6]. Since this study was conducted in patients with TNBC who underwent PORT at a time when treatments such as immunotherapy and targeted therapy were not actively applied in South Korea, multicenter clinical studies that include patients to whom such treatments were applied are needed.

This study had several limitations. First, this was a retrospective study, which is prone to selection bias even in a multicenter study. Second, the number of patients and observed events were small. Lastly, given the widespread adoption of neoadjuvant chemotherapy and targeted therapy, even though immunotherapy has also been demonstrated to improve overall survival and response in TNBC [2], neoadjuvant chemotherapy was performed only in 18.6% of patients, and no patient underwent targeted therapy or immunotherapy in this study. Nevertheless, this study is meaningful as a multicenter study on TNBC in South Korea, although it was retrospective.

## 5. Conclusions

We verified that T and N stages, *LVI*, and *Ki-67* expression were significantly related to survival outcomes after postoperative radiation therapy in patients with TNBC in South Korea. Distant metastasis was the main treatment failure on TNBC. Multi-institutional clinical studies including patients with TNBC who have undergone immunotherapy or targeted therapy are needed in South Korea.

## Figures and Tables

**Table 1:** Baseline characteristics.

Characteristics		Number	(%)
Age (year, median, range)		49	(range: 24–80)
Pathology			
	IDC	622	(88.9)
	Metaplastic	23	(3.3)
	medullary	13	(1.9)
	Apocrine	11	(1.6)
	DCIS	10	(1.4)
	microinvasive	7	(1.0)
	Others	13	(1.9)
Histologic grade			
	1	10	(1.4)
	2	103	(14.7)
	3	538	(77.0)
	unknown	48	(6.9)
Lympho-vascular invasion			
	positive	240	(34.3)
	negative	435	(62.3)
	unknown	24	(3.4)
Extracapsular extension			
	positive	49	(7.0)
	negative	557	(79.7)
	unknown	93	(13.3)
*Ki-67*			
	negative	3	(0.4)
	≤5%	131	(18.7)
	6–25%	211	(30.2)
	>25%	198	(28.3)
	unknown	156	(22.3)
T stage			
	in situ	3	(0.4)
	1	268	(38.3)
	2	355	(50.8)
	3	61	(8.7)
	unknown	12	(17.1)
N stage			
	positive	254	(36.3)
	negative	443	(63.4)
	unknown	2	(0.3)
Stage			
	0	3	(0.4)
	I	216	(30.9)
	IIA	270	(38.6)
	IIB	110	(15.7)
	III	100	(14.3)

N, nodal; T, tumor.

**Table 2:** Treatments.

Characteristics		Number	(%)
Neoadjuvant chemotherapy			
	Yes	130	(18.6)
	No	569	(82.4)
Neoadjuvant chemotherapy regimen			
	AC	92	(70.8)
	AC-T	26	(20.0)
	other	12	(9.2)
Surgery			
	BCS only	1	(0.1)
	BCS and SLNB	329	(47.1)
	BCS and ALND	304	(43.5)
	Mastectomy and SLNB	1	(0.1)
	Mastectomy and ALNB	64	(9.2)
Radiotherapy dose (mainly)			
	Breast/chest wall	45–50.4 Gy in 25–28 fractions	
	Tumor bed boost	10–16 Gy in 5–8 fractions	
	SCLN/IMN	45–50 Gy in 25 fractions	
Adjuvant chemotherapy			
	Yes	596	(85.3)
	No	103	(14.7)
Adjuvant regimen			
	FAC	259	(43.5)
	AC	118	(19.8)
	AC-T	68	(11.4)
	AC-D	46	(7.7)
	CMF	44	(7.4)
	DA	35	(5.9)
	other	26	(4.3)

AC, Adriamycin cyclophosphamide; AC-D, Adriamycin cyclophosphamide docetaxel; AC-T, Adriamycin-cyclophosphamide-paclitaxel; ALND, axillary lymph node dissection; BCS, breast-conserving surgery; CMF, cyclophosphamide-methotrexate-5-fluorouracil; DA, docetaxel-Adriamycin; FAC, 5-fluorouracil Adriamycin cyclophosphamide; IMN, internal mammary lymph node; SCLN, supraclavicular lymph node; SLNB, sentinel lymph node biopsy.

**Table 3:** Univariate analysis of prognostic factors related to survival rates.

Characteristics	No. of Patient	OS		LRRFS		DMFS		DFS		CSS	
		5 yr (%)	*p*	5 yr (%)	*p*	5 yr (%)	*p*	5 yr (%)	*p*	5 yr (%)	*p*
Age			0.16		NS		0.01		0.048		NS
50 or younger	404	90.5		91.3		87.1		82.6		90.8	
Older than 50	295	92.9		93.6		92.7		88.8		93.3	
T stage			<0.001		<0.001		<0.001		<0.001		<0.001
T1	268	96.6		96.6		95.0		93.2		96.6	
T2	355	90.7		91.8		88.1		83.6		90.9	
T3	61	75.1		77.1		71.0		59.3		75.1	
N stage			<0.001		<0.001		<0.001		<0.001		<0.001
Negative	443	94.9		94.9		93.3		90.1		95.1	
Positive	254	85.5		87.6		82.5		76.5		85.8	
Grade			0.01		NS		0.051		0.091		0.014
Low	113	96.3		96.3		93.6		90.0		96.3	
High	538	92.7		91.9		88.2		84.3		90.4	
LVI			0.04		0.01		<0.001		<0.001		0.046
Negative	435	93.2		94.5		92.7		89.4		93.6	
Positive	240	87.6		88.8		82.9		77.6		88.1	
ECE			<0.001		<0.001		<0.001		<0.001		<0.001
Negative	557	92.4		93.3		91.9		88.1		92.6	
Positive	49	74.4		77.1		60.3		50.1		74.4	
*Ki-67*			0.036		0.016		0.041		0.002		0.027
≤5%	134	94.7		97.7		93.8		93.1		95.4	
>5%	410	88.2		89.1		85.4		80.3		88.7	

5 yr, five years; CSS, cause-specific survival; DFS, disease-free survival; DMFS, distant metastasis-free survival; ECE, extracapsular extension; LRRFS, loco-regional recurrence survival; LVI, lympho-vascular invasion, N, nodal; No, number; NS, nonspecific; OS, overall survival; P, probability; T, tumor.

**Table 4:** Multivariate analysis of prognostic factors related to survival rates.

Characteristics	No. of Patient	OS		LRRFS		DMFS		DFS		CSS	
		HR (95% CI)	*p*	HR (95% CI)	*p*	HR (95% CI)	*p*	HR (95% CI)	*p*	HR (95% CI)	*p*
T stage			0.003		0.001		0.014		0.000		0.006
T1	268	1		1		1		1		1	
T2	355	1.856 (1.213–2.84)		2.203 (1.378–3.523)		1.649 (1.106–2.457)		1.991 (1.403–2.825)		1.839 (1.194–2.832)	
T3	61										
N stage			0.004		0.026		0.002		0.001		0.001
Negative	443	1 2.551 (1.460–4.459)		1 1.966 (1.085–3.564)		1 2.287 (1.347–3.884)		1 2.132 (1.360–3.341)		1 2.606 (1.474–4.605)	
Positive	254										
LVI					0.044		0.05				
Negative	435			1		0.600 (0.360–1.000)					
Positive	240			1.227 (1.006–1.496)		1					
*Ki-67*					0.015				0.019		
≤5%	134			1				1			
>5%	410			3.148 (1.246–7.955)				2.188 (1.140–4.197)			

CI, confidence interval; CSS, cause-specific survival; DFS, disease-free survival; DMFS, distant metastasis-free survival; HR, hazard ratio; LRRFS, loco-regional recurrence survival; LVI, lympho-vascular invasion; OS, overall survival; N, nodal; No, numbers; T, tumor.

## Data Availability

The original contributions presented in the study are included in the article, further inquiries can be directed to the corresponding authors.
